# (±)-Ethyl 6,7-dimeth­oxy-1-(1*H*-pyrrol-2-yl)-1,2,3,4-tetra­hydroisoquinoline-2-car­boxyl­ate

**DOI:** 10.1107/S1600536808026020

**Published:** 2008-08-20

**Authors:** Rosica Petrova Nikolova, Tsonko Kolev, Stela M. Statkova-Abeghe, Boris Lubomirov Shivachev

**Affiliations:** a Department of Advanced Materials Science and Engineering, Faculty of Engineering, Yamaguchi University, 2-16-1 Tokiwadai, Ube 755-8611, Japan; b Bulgarian Academy of Sciences, Institute of Organic Chemistry, Acad G. Bonchev Str. build. 9, 1113 Sofia, Bulgaria; c Plovdiv University, Department of Organic Chemistry, 4000 Plovdiv, Bulgaria; d Department of Structural Biology, University of Pittsburgh School of Medicine, 3501 5th Ave., Pittsburgh 15260, USA

## Abstract

In the title compound, C_18_H_22_N_2_O_4_, the dihedral angle between the pyrrolyl and quinolinyl fragments is 68.97 (2)°. Two non-classical intra­molecular C—H⋯O hydrogen bonds stabilize the mol­ecular geometry. In the crystal structure, mol­ecules form infinite chains *via* moderate inter­molecular N—H⋯O(CH_3_) hydrogen bonds.

## Related literature

For related crystal structures, see: Kolev *et al.* (2007 ▶); Petrova *et al.* (2007 ▶); Petrova *et al.* (2005 ▶); Rajnikant *et al.* (2002 ▶); Shishkina *et al.* (2005 ▶); Venkov *et al.* (2004 ▶); Vincente *et al.* (2005 ▶).
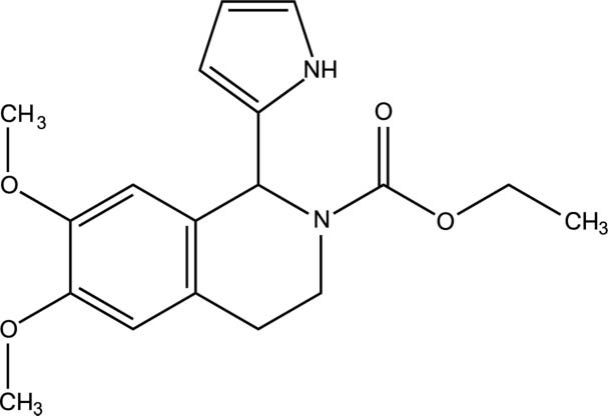


## Experimental

### 

#### Crystal data

C_18_H_22_N_2_O_4_
*M* *_r_* = 330.38
Monoclinic, 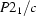

*a* = 8.403 (3) Å
*b* = 17.046 (3) Å
*c* = 11.6486 (13) Å
β = 95.260 (13)°
*V* = 1661.5 (7) Å^3^
*Z* = 4
Mo *K*α radiation
μ = 0.09 mm^−1^
*T* = 290 (2) K
0.32 × 0.32 × 0.30 mm

#### Data collection

Enraf–Nonius CAD-4 diffractometer
Absorption correction: none
6852 measured reflections
3263 independent reflections
1828 reflections with *I* > 2σ(*I*)
*R*_int_ = 0.110
3 standard reflections frequency: 120 min intensity decay: none

#### Refinement

*R*[*F* ^2^ > 2σ(*F* ^2^)] = 0.065
*wR*(*F* ^2^) = 0.156
*S* = 1.07
3263 reflections
218 parameters
H-atom parameters constrained
Δρ_max_ = 0.21 e Å^−3^
Δρ_min_ = −0.20 e Å^−3^

### 

Data collection: *CAD-4 EXPRESS* (Enraf–Nonius, 1994 ▶); cell refinement: *CAD-4 EXPRESS*; data reduction: *XCAD4* (Harms & Wocadlo, 1995 ▶); program(s) used to solve structure: *SHELXS97* (Sheldrick, 2008 ▶); program(s) used to refine structure: *SHELXL97* (Sheldrick, 2008 ▶); molecular graphics: *ORTEP-3 for Windows* (Farrugia, 1997 ▶) and *Mercury* (Macrae *et al.*, 2006 ▶); software used to prepare material for publication: *WinGX* (Farrugia, 1999 ▶).

## Supplementary Material

Crystal structure: contains datablocks I, global. DOI: 10.1107/S1600536808026020/pv2093sup1.cif

Structure factors: contains datablocks I. DOI: 10.1107/S1600536808026020/pv2093Isup2.hkl

Additional supplementary materials:  crystallographic information; 3D view; checkCIF report

## Figures and Tables

**Table 1:** Hydrogen-bond geometry (Å, °)

*D*—H⋯*A*	*D*—H	H⋯*A*	*D*⋯*A*	*D*—H⋯*A*
N3—H3⋯O1^i^	0.86	2.49	3.225 (4)	145
N3—H3⋯O2^i^	0.86	2.38	3.018 (4)	132
C7—H7⋯O4	0.98	2.34	2.784 (4)	107
C8—H8*B*⋯O3	0.97	2.29	2.653 (4)	101

Symmetry code: (i) 
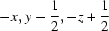
.

**Fig. 1.**
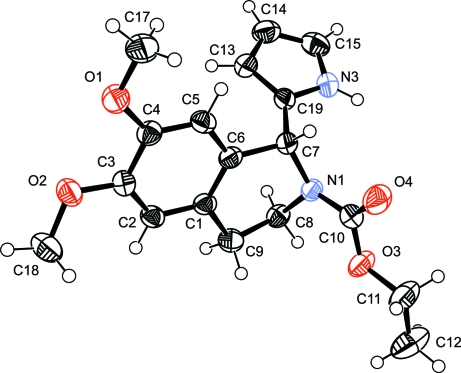
View of the structure and the atom-numbering scheme of (I) showing 50% probability displacement ellipsoids. H atoms are shown as small spheres of arbitrary radii.

**Fig. 2.**
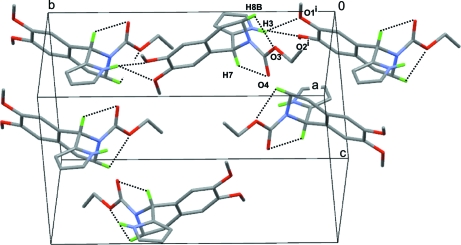
A view of the molecular packing in (I). Hydrogen bonds are represented by dotted lines. H atoms not involved in hydrogen bonding interactions have been omitted. [Symmetry code: (i) -x, -1/2 + y, 1/2 - z].

## supplementary crystallographic information

### Comment

As part of our research program on tetrahydroisoquinolines (Kolev *et al.*, 2007; Petrova *et al.*, 2007; Petrova *et al.*, 2005) the crystal structure of the title compound,(I), has been solved. The molecule possesses regular geometry with two nearly planar ring systems. The r.m.s. deviation of pyrrolyl and quinolin-2(1*H*)-fragments is 0.161 (7) Å and 0.002 (2) Å, respectively, and the dihedral angle between their mean planes is 68.97 (2)°. The geometrical parameters of both rings are comparable to those observed in other quinoline derivatives (Rajnikant *et al.*, 2002; Vincente *et al.*, 2005; Shishkina *et al.*, 2005). Two non-classsical intramolecular hydrogen bonds (C7—H7···O4 and C8—H8···O3) stabilize the molecular geometry. Only the methoxy O atoms are realised as hydrogen bond acceptors and together with the only possible donor form a bifurcated hydrogen bond of the N—H···(O,*O*) type. Thus neighboring molecules are oriented head-to-tail and connected to form infinite chains along the *b*-axis (Fig. 2).

### Experimental

The title compound has been obtained following the procedure described by Venkov *et al.*, 2004. Colorless crystals of (I) suitable for X-ray diffraction were obtained by slow evaporation from ethanol/water (2:1) solution.

### Refinement

All H atoms were placed in idealized positions (*C*—H_methyl_ = 0.96 Å, *C*—H_methylen_ = 0.97 Å, C—H_aromatic_ = 0.93Å and N—H = 0.86 Å) and were constrained to ride on their parent atoms, with *U*_iso_(H) = 1.5*U*_eq_(C_methyl_) or *U*_iso_(H) = 1.2*U*_eq_(C_aromatic_, C_methylen_ ~ or N). The high *R*_int_ value (0.11) and relatively low ratio (0.55) of observed to unique reflections may be a result of the poor diffraction quality of the crystal.

### Crystal data

**Table:**

C_18_H_22_N_2_O_4_	*F*_000_ = 704
*M**_r_* = 330.38	*D*_x_ = 1.321 Mg m^−^^3^
Monoclinic, *P*2_1_/*c*	Mo *K*α radiation λ = 0.71073 Å
Hall symbol: -P 2ybc	Cell parameters from 22 reflections
*a* = 8.403 (3) Å	θ = 18.3–18.8º
*b* = 17.046 (3) Å	µ = 0.09 mm^−^^1^
*c* = 11.6486 (13) Å	*T* = 290 (2) K
β = 95.260 (13)º	Prism, colorless
*V* = 1661.5 (7) Å^3^	0.32 × 0.32 × 0.30 mm
*Z* = 4	

### Data collection

**Table:**

Enraf–Nonius CAD-4 diffractometer	*R*_int_ = 0.110
Radiation source: fine-focus sealed tube	θ_max_ = 26.0º
Monochromator: graphite	θ_min_ = 2.1º
*T* = 290(2) K	*h* = 0→10
Nonprofiled ω/2θ scans	*k* = −20→20
Absorption correction: none	*l* = −14→14
6852 measured reflections	3 standard reflections
3263 independent reflections	every 120 min
1828 reflections with *I* > 2σ(*I*)	intensity decay: −1%

### Refinement

**Table:**

Refinement on *F*^2^	Hydrogen site location: inferred from neighbouring sites
Least-squares matrix: full	H-atom parameters constrained
*R*[*F*^2^ > 2σ(*F*^2^)] = 0.065	*w* = 1/[σ^2^(*F*_o_^2^) + (0.0293*P*)^2^ + 2.0348*P*] where *P* = (*F*_o_^2^ + 2*F*_c_^2^)/3
*wR*(*F*^2^) = 0.156	(Δ/σ)_max_ < 0.001
*S* = 1.07	Δρ_max_ = 0.21 e Å^−^^3^
3263 reflections	Δρ_min_ = −0.20 e Å^−^^3^
218 parameters	Extinction correction: SHELXL97 (Sheldrick, 2008), Fc^*^=kFc[1+0.001xFc^2^λ^3^/sin(2θ)]^-1/4^
Primary atom site location: structure-invariant direct methods	Extinction coefficient: 0.0075 (10)
Secondary atom site location: difference Fourier map	

### Special details

**Table:**

Geometry. All e.s.d.'s (except the e.s.d. in the dihedral angle between two l.s. planes) are estimated using the full covariance matrix. The cell e.s.d.'s are taken into account individually in the estimation of e.s.d.'s in distances, angles and torsion angles; correlations between e.s.d.'s in cell parameters are only used when they are defined by crystal symmetry. An approximate (isotropic) treatment of cell e.s.d.'s is used for estimating e.s.d.'s involving l.s. planes.
Refinement. Refinement of *F*^2^ against ALL reflections. The weighted *R*-factor *wR* and goodness of fit *S* are based on *F*^2^, conventional *R*-factors *R* are based on *F*, with *F* set to zero for negative *F*^2^. The threshold expression of *F*^2^ > σ(*F*^2^) is used only for calculating *R*-factors(gt) *etc*. and is not relevant to the choice of reflections for refinement. *R*-factors based on *F*^2^ are statistically about twice as large as those based on *F*, and *R*- factors based on ALL data will be even larger.

### Fractional atomic coordinates and isotropic or equivalent isotropic displacement parameters (Å^2^)

**Table:**

	*x*	*y*	*z*	*U*_iso_*/*U*_eq_	
O1	0.2084 (3)	0.63581 (14)	0.3413 (2)	0.0474 (7)	
O2	0.3254 (3)	0.65266 (14)	0.1467 (2)	0.0443 (7)	
O3	0.2237 (3)	0.20530 (14)	0.1157 (2)	0.0476 (7)	
O4	0.2206 (3)	0.23765 (16)	0.3033 (2)	0.0573 (8)	
N1	0.0630 (3)	0.30217 (16)	0.1633 (2)	0.0336 (7)	
C2	0.2399 (4)	0.5223 (2)	0.0832 (3)	0.0339 (8)	
H2	0.2815	0.5290	0.0126	0.041*	
C19	−0.1547 (4)	0.37433 (19)	0.2412 (3)	0.0339 (8)	
C6	0.1037 (4)	0.44139 (19)	0.2113 (3)	0.0329 (8)	
C3	0.2542 (4)	0.58174 (19)	0.1623 (3)	0.0337 (8)	
C13	−0.2492 (4)	0.4391 (2)	0.2366 (3)	0.0415 (9)	
H13	−0.2154	0.4906	0.2277	0.050*	
C7	0.0237 (4)	0.36515 (18)	0.2422 (3)	0.0347 (8)	
H7	0.0659	0.3503	0.3204	0.042*	
C9	0.1515 (4)	0.3882 (2)	0.0146 (3)	0.0408 (9)	
H9A	0.2564	0.3657	0.0085	0.049*	
H9B	0.1157	0.4115	−0.0593	0.049*	
C5	0.1184 (4)	0.5026 (2)	0.2922 (3)	0.0352 (8)	
H5	0.0780	0.4956	0.3632	0.042*	
N3	−0.2512 (3)	0.31109 (17)	0.2555 (2)	0.0418 (8)	
H3	−0.2197	0.2631	0.2616	0.050*	
C10	0.1731 (4)	0.2482 (2)	0.2028 (3)	0.0395 (9)	
C4	0.1907 (4)	0.5724 (2)	0.2694 (3)	0.0349 (8)	
C18	0.4219 (5)	0.6587 (2)	0.0526 (3)	0.0555 (11)	
H18A	0.4648	0.7109	0.0499	0.083*	
H18B	0.3580	0.6479	−0.0182	0.083*	
H18C	0.5080	0.6216	0.0626	0.083*	
C1	0.1642 (4)	0.45151 (19)	0.1057 (3)	0.0323 (8)	
C15	−0.4056 (5)	0.3361 (2)	0.2588 (3)	0.0498 (10)	
H15	−0.4936	0.3042	0.2672	0.060*	
C8	0.0366 (4)	0.3237 (2)	0.0414 (3)	0.0356 (8)	
H8A	−0.0725	0.3415	0.0240	0.043*	
H8B	0.0529	0.2782	−0.0061	0.043*	
C17	0.1649 (5)	0.6268 (2)	0.4555 (3)	0.0527 (11)	
H17A	0.1816	0.6754	0.4965	0.079*	
H17B	0.2294	0.5865	0.4941	0.079*	
H17C	0.0543	0.6123	0.4532	0.079*	
C14	−0.4078 (5)	0.4148 (2)	0.2476 (3)	0.0491 (10)	
H14	−0.4971	0.4471	0.2471	0.059*	
C11	0.3387 (6)	0.1452 (3)	0.1464 (4)	0.0675 (14)	
H11A	0.2972	0.1094	0.2010	0.081*	
H11B	0.4361	0.1685	0.1824	0.081*	
C12	0.3725 (6)	0.1029 (3)	0.0433 (4)	0.0812 (16)	
H12A	0.4508	0.0630	0.0633	0.122*	
H12B	0.4129	0.1387	−0.0106	0.122*	
H12C	0.2761	0.0790	0.0090	0.122*	

### Atomic displacement parameters (Å^2^)

**Table:**

	*U*^11^	*U*^22^	*U*^33^	*U*^12^	*U*^13^	*U*^23^
O1	0.0666 (18)	0.0391 (15)	0.0374 (14)	−0.0089 (13)	0.0099 (13)	−0.0089 (12)
O2	0.0514 (15)	0.0414 (15)	0.0412 (14)	−0.0117 (12)	0.0110 (12)	−0.0012 (12)
O3	0.0551 (17)	0.0418 (15)	0.0455 (15)	0.0201 (13)	0.0028 (13)	−0.0023 (13)
O4	0.073 (2)	0.0578 (18)	0.0403 (16)	0.0254 (15)	0.0029 (14)	0.0079 (14)
N1	0.0403 (17)	0.0308 (16)	0.0298 (14)	0.0073 (14)	0.0035 (13)	0.0008 (13)
C2	0.0360 (19)	0.039 (2)	0.0275 (17)	0.0023 (16)	0.0060 (15)	0.0038 (15)
C19	0.043 (2)	0.0306 (19)	0.0297 (18)	−0.0011 (16)	0.0094 (16)	0.0005 (15)
C6	0.0326 (19)	0.0323 (19)	0.0339 (18)	0.0051 (15)	0.0040 (16)	−0.0011 (15)
C3	0.0330 (19)	0.0326 (19)	0.0351 (19)	−0.0019 (15)	0.0009 (16)	0.0030 (16)
C13	0.046 (2)	0.037 (2)	0.043 (2)	0.0064 (18)	0.0088 (18)	0.0034 (17)
C7	0.044 (2)	0.0297 (18)	0.0317 (18)	0.0025 (16)	0.0092 (16)	0.0011 (15)
C9	0.047 (2)	0.042 (2)	0.0335 (19)	0.0006 (18)	0.0074 (18)	−0.0034 (17)
C5	0.037 (2)	0.041 (2)	0.0289 (17)	0.0009 (17)	0.0065 (15)	0.0007 (17)
N3	0.0453 (19)	0.0350 (17)	0.0460 (18)	−0.0009 (15)	0.0084 (15)	0.0054 (15)
C10	0.043 (2)	0.037 (2)	0.039 (2)	0.0001 (18)	0.0094 (18)	0.0029 (17)
C4	0.041 (2)	0.0339 (19)	0.0297 (18)	0.0008 (16)	0.0014 (16)	−0.0043 (16)
C18	0.053 (3)	0.064 (3)	0.052 (2)	−0.016 (2)	0.016 (2)	0.000 (2)
C1	0.0322 (19)	0.0352 (19)	0.0299 (17)	0.0062 (15)	0.0043 (15)	0.0019 (15)
C15	0.041 (2)	0.060 (3)	0.050 (2)	−0.009 (2)	0.0124 (19)	−0.001 (2)
C8	0.041 (2)	0.0345 (19)	0.0306 (18)	0.0032 (16)	−0.0010 (15)	−0.0055 (16)
C17	0.067 (3)	0.054 (3)	0.038 (2)	−0.003 (2)	0.009 (2)	−0.011 (2)
C14	0.044 (2)	0.057 (3)	0.047 (2)	0.013 (2)	0.009 (2)	0.000 (2)
C11	0.077 (3)	0.052 (3)	0.073 (3)	0.030 (2)	0.006 (3)	−0.004 (2)
C12	0.083 (4)	0.076 (3)	0.084 (4)	0.035 (3)	0.004 (3)	−0.023 (3)

### Geometric parameters (Å, °)

**Table:**

O1—C4	1.367 (4)	C9—C8	1.515 (5)
O1—C17	1.420 (4)	C9—H9A	0.9700
O2—C3	1.369 (4)	C9—H9B	0.9700
O2—C18	1.426 (4)	C5—C4	1.374 (5)
O3—C10	1.350 (4)	C5—H5	0.9300
O3—C11	1.431 (5)	N3—C15	1.369 (5)
O4—C10	1.216 (4)	N3—H3	0.8600
N1—C10	1.355 (4)	C18—H18A	0.9600
N1—C8	1.464 (4)	C18—H18B	0.9600
N1—C7	1.470 (4)	C18—H18C	0.9600
C2—C3	1.367 (5)	C15—C14	1.349 (5)
C2—C1	1.399 (4)	C15—H15	0.9300
C2—H2	0.9300	C8—H8A	0.9700
C19—C13	1.359 (5)	C8—H8B	0.9700
C19—N3	1.368 (4)	C17—H17A	0.9600
C19—C7	1.506 (5)	C17—H17B	0.9600
C6—C1	1.385 (4)	C17—H17C	0.9600
C6—C5	1.403 (4)	C14—H14	0.9300
C6—C7	1.521 (4)	C11—C12	1.451 (6)
C3—C4	1.410 (4)	C11—H11A	0.9700
C13—C14	1.413 (5)	C11—H11B	0.9700
C13—H13	0.9300	C12—H12A	0.9600
C7—H7	0.9800	C12—H12B	0.9600
C9—C1	1.510 (4)	C12—H12C	0.9600
			
C4—O1—C17	117.8 (3)	O1—C4—C5	126.3 (3)
C3—O2—C18	116.9 (3)	O1—C4—C3	115.1 (3)
C10—O3—C11	116.9 (3)	C5—C4—C3	118.6 (3)
C10—N1—C8	122.5 (3)	O2—C18—H18A	109.5
C10—N1—C7	118.0 (3)	O2—C18—H18B	109.5
C8—N1—C7	113.6 (3)	H18A—C18—H18B	109.5
C3—C2—C1	121.8 (3)	O2—C18—H18C	109.5
C3—C2—H2	119.1	H18A—C18—H18C	109.5
C1—C2—H2	119.1	H18B—C18—H18C	109.5
C13—C19—N3	107.1 (3)	C6—C1—C2	118.9 (3)
C13—C19—C7	131.5 (3)	C6—C1—C9	121.8 (3)
N3—C19—C7	121.2 (3)	C2—C1—C9	119.3 (3)
C1—C6—C5	119.2 (3)	C14—C15—N3	108.2 (3)
C1—C6—C7	121.5 (3)	C14—C15—H15	125.9
C5—C6—C7	119.3 (3)	N3—C15—H15	125.9
O2—C3—C2	125.3 (3)	N1—C8—C9	109.8 (3)
O2—C3—C4	115.0 (3)	N1—C8—H8A	109.7
C2—C3—C4	119.7 (3)	C9—C8—H8A	109.7
C19—C13—C14	108.1 (3)	N1—C8—H8B	109.7
C19—C13—H13	125.9	C9—C8—H8B	109.7
C14—C13—H13	125.9	H8A—C8—H8B	108.2
N1—C7—C19	110.6 (3)	O1—C17—H17A	109.5
N1—C7—C6	110.3 (2)	O1—C17—H17B	109.5
C19—C7—C6	111.7 (3)	H17A—C17—H17B	109.5
N1—C7—H7	108.0	O1—C17—H17C	109.5
C19—C7—H7	108.0	H17A—C17—H17C	109.5
C6—C7—H7	108.0	H17B—C17—H17C	109.5
C1—C9—C8	112.3 (3)	C15—C14—C13	107.2 (3)
C1—C9—H9A	109.1	C15—C14—H14	126.4
C8—C9—H9A	109.1	C13—C14—H14	126.4
C1—C9—H9B	109.1	O3—C11—C12	109.2 (4)
C8—C9—H9B	109.1	O3—C11—H11A	109.8
H9A—C9—H9B	107.9	C12—C11—H11A	109.8
C4—C5—C6	121.8 (3)	O3—C11—H11B	109.8
C4—C5—H5	119.1	C12—C11—H11B	109.8
C6—C5—H5	119.1	H11A—C11—H11B	108.3
C19—N3—C15	109.4 (3)	C11—C12—H12A	109.5
C19—N3—H3	125.3	C11—C12—H12B	109.5
C15—N3—H3	125.3	H12A—C12—H12B	109.5
O4—C10—O3	123.1 (3)	C11—C12—H12C	109.5
O4—C10—N1	125.5 (3)	H12A—C12—H12C	109.5
O3—C10—N1	111.4 (3)	H12B—C12—H12C	109.5
			
C18—O2—C3—C2	−14.4 (5)	C8—N1—C10—O3	−14.8 (5)
C18—O2—C3—C4	166.3 (3)	C7—N1—C10—O3	−166.0 (3)
C1—C2—C3—O2	−179.6 (3)	C17—O1—C4—C5	7.6 (5)
C1—C2—C3—C4	−0.4 (5)	C17—O1—C4—C3	−172.4 (3)
N3—C19—C13—C14	−0.4 (4)	C6—C5—C4—O1	178.8 (3)
C7—C19—C13—C14	−175.2 (4)	C6—C5—C4—C3	−1.2 (5)
C10—N1—C7—C19	−132.3 (3)	O2—C3—C4—O1	0.6 (5)
C8—N1—C7—C19	74.0 (3)	C2—C3—C4—O1	−178.7 (3)
C10—N1—C7—C6	103.6 (3)	O2—C3—C4—C5	−179.4 (3)
C8—N1—C7—C6	−50.1 (4)	C2—C3—C4—C5	1.3 (5)
C13—C19—C7—N1	−135.5 (4)	C5—C6—C1—C2	0.7 (5)
N3—C19—C7—N1	50.3 (4)	C7—C6—C1—C2	−178.5 (3)
C13—C19—C7—C6	−12.3 (5)	C5—C6—C1—C9	−179.6 (3)
N3—C19—C7—C6	173.6 (3)	C7—C6—C1—C9	1.2 (5)
C1—C6—C7—N1	16.3 (5)	C3—C2—C1—C6	−0.6 (5)
C5—C6—C7—N1	−162.9 (3)	C3—C2—C1—C9	179.7 (3)
C1—C6—C7—C19	−107.1 (4)	C8—C9—C1—C6	12.8 (5)
C5—C6—C7—C19	73.7 (4)	C8—C9—C1—C2	−167.5 (3)
C1—C6—C5—C4	0.2 (5)	C19—N3—C15—C14	−0.5 (4)
C7—C6—C5—C4	179.4 (3)	C10—N1—C8—C9	−86.6 (4)
C13—C19—N3—C15	0.6 (4)	C7—N1—C8—C9	65.8 (4)
C7—C19—N3—C15	176.0 (3)	C1—C9—C8—N1	−44.0 (4)
C11—O3—C10—O4	0.0 (5)	N3—C15—C14—C13	0.2 (5)
C11—O3—C10—N1	−179.1 (3)	C19—C13—C14—C15	0.1 (4)
C8—N1—C10—O4	166.2 (4)	C10—O3—C11—C12	177.0 (4)
C7—N1—C10—O4	15.0 (5)		

### Hydrogen-bond geometry (Å, °)

**Table:**

*D*—H···*A*	*D*—H	H···*A*	*D*···*A*	*D*—H···*A*
N3—H3···O1^i^	0.86	2.49	3.225 (4)	145
N3—H3···O2^i^	0.86	2.38	3.018 (4)	132
C7—H7···O4	0.98	2.34	2.784 (4)	107
C8—H8B···O3	0.97	2.29	2.653 (4)	101

Symmetry codes: (i) −*x*, *y*−1/2, −*z*+1/2.
